# The Medical Era

**Published:** 1883-12

**Authors:** 


### NEW JOURNALS.

THE MEDICAL ERA was started some months ago in Chicago. Dr. James E. Gross is the editor, assisted by a goodly number of able contributors.

The number before us is exceedingly well gotten up and is very interesting. Of Homœopathy there does not appear to be much; but *that* is not now a popular article to offer for sale. The publishers announce that the *Era* has "come to stay." We are glad to hear this in this mutable world; it is pleasant to feel that one *Era* will be immutable! "Financially, *The Medical Era* is solid." A second cause for gratification! But look out for one Jay Gould, my friends. He is said to have an eye for financially solid institutions.

